# Molecular characterization of cryptic and sympatric lymnaeid species from the *Galba/Fossaria* group in Mendoza Province, Northern Patagonia, Argentina

**DOI:** 10.1186/1756-3305-6-304

**Published:** 2013-10-23

**Authors:** Claire J Standley, Lucila Prepelitchi, Silvia M Pietrokovsky, Laura Issia, J Russell Stothard, Cristina Wisnivesky-Colli

**Affiliations:** 1 Ecology & Evolutionary Biology, Princeton University, Princeton, NJ 08544, USA; 2 Unidad de Ecología de Reservorios y Vectores de Parásitos, Departamento de Ecología, Genética y Evolución, Facultad de Ciencias Exactas y Naturales, Universidad de Buenos Aires, Ciudad Universitaria, Pabellón 2, 4 piso, Laboratorio 55, Ciudad Autónoma de Buenos Aires, C1428EGA, Argentina; 3 Consejo Nacional de Investigaciones Científicas y Técnicas, Av. Rivadavia 1917, Ciudad Autónoma de Buenos Aires 1033, Argentina; 4 Department of Parasitology, Liverpool School of Tropical Medicine, Pembroke Place, Liverpool L3 5QA, UK

**Keywords:** Population genetics, Taxonomy, Freshwater lymnaeid snails, *Galba*, Fascioliasis, Northern Patagonia, Argentina

## Abstract

**Background:**

Freshwater lymnaeid snails can act as the intermediate hosts for trematode parasites such as the liver fluke *Fasciola hepatica*, that cause significant economic and biomedical burden worldwide, particularly through bovine fascioliasis. Transmission potential is tightly coupled to local compatibility with snail hosts, so accurate identification of lymnaeid species is crucial for understanding disease risk, especially when invasive species are encountered. Mendoza Province, in Argentina, is a center of livestock production and also an area of endemic fascioliasis transmission. However, the distribution of lymnaeid species in the region is not well known.

**Methods:**

This study examined lymnaeid snails from seven localities in the Department of Malarguë, Mendoza Province, using morphological and molecular analyses and also describing ecological variables associated with snail presence.

**Results:**

While morphological characters identified two species of lymnaeid, *Galba truncatula* and *G. viatrix,* molecular data revealed a third, cryptic species, *G. neotropica*, which was sympatric with *G. viatrix*. *G. truncatula* was exclusively found in high altitude (>1900 meters above sea level [masl]) sites, whereas mixed *G. neotropica/G. viatrix* localities were at middle elevations (1300–1900 masl), and *G. viatrix* was found alone at the lowest altitude sites (<1300 masl). Phylogenetic analysis using two mitochondrial markers revealed *G. neotropica* and *G. viatrix* to be closely related, and given their morphological similarities, their validities as separate taxonomic entities should be questioned.

**Conclusions:**

This study highlights the need of a robust taxonomic framework for the identification of lymnaeid snails, incorporating molecular, morphological and ecological variables while avoiding nomenclature redundancy. As the three species observed here, including one alien invasive species, are considered hosts of varying susceptibility to *Fasciola* parasites, and given the economic importance of fascioliasis for livestock production, this research has critical importance for the ultimate aim of controlling disease transmission.

## Background

Freshwater lymnaeid snails act as the intermediate hosts for various parasites, including the liver flukes *Fasciola hepatica* and *F. gigantica*, which cause fascioliasis (also referred to as fasciolosis) in livestock and also in humans around the world [1]. The disease can confer large economic losses on livestock-based economies, due to liver condemnation, decreases in milk, wool and meat production and also increased mortality [2]. The compatibility between *Fasciola* spp. and their intermediate host snails is tightly coupled [3]; as such, the distribution of suitable snail species is an important factor in determining the geographical extent of fascioliasis transmission. Moreover, the biological characteristics of these snails are one of the most important controlling factors determining and shaping transmission [3]. However, a research focus is needed on the construction of a reliable identification system and a solid phylogenetic framework for lymnaeid snails, which could help in the definition of areas with high fascioliasis epidemiological risk [4].

Lymnaeid taxonomy is confused and presently confusing, with numerous different genera, many redundancies in the nomenclature and constant revisions in species definitions [5,6]. Contributing to this taxonomic problem is the variability of morphological characters traditionally used to determine species identifications, such as the characteristics of the shell, radula, renal organ and male reproductive organs [7-9]. Shell characters are notoriously plastic, and can often vary considerably within a species according to environmental conditions or other external forces [10,11]. Internal morphological characters, specifically the reproductive structures, are considered more robust for species identification, although in some cases, they fail to distinguish between species with marked intraspecific morphological variability (sibling species) or variants within a species [12]. In recent years there has been a movement towards molecular-based tools as additional diagnostic protocols for this taxonomically difficult yet biomedically important snail group [13,14].

Recent molecular work has identified a handful of genetic regions, both in the mitochondrial and nuclear genomes, which are suitable for species as well as population-level analyses [15-18]. These markers have greatly elucidated our understanding of the biogeography and distribution of fascioliasis intermediate hosts, particularly in South America, where its economic impact on livestock production is significant [19,20]. Many mitochondrial and nuclear markers (ITS-1, 16S, COI, etc.) have been used to clarify the taxonomic status within the cryptic South American lymnaeids, namely *Galba* (= *Lymnaea*) *truncatula, Galba* (= *Lymnaea*) *viatrix, Galba* (= *Lymnaea*) *neotropica* and *Galba* (= *Lymnaea*) *cubensis*[4,13].

Of these, *G. viatrix* and *G. truncatula* have been found inhabiting different geographic areas in Argentina [21-23]. In the endemic fascioliasis zone of Malargüe, Mendoza province, Northern Patagonia, both snail species were found in Wildlife Reserves and were morphologically identified [19,23]. Based on the same surveys, the concomitant presence of *F. hepatica* infection in *G. truncatula*, *G. viatrix* and mammals, suggested the existence of a permanent transmission foci in the area [19,23]. The *F. hepatica* prevalence in snails was estimated as 2% [23] whereas that in domestic and wild herbivores was observed as 84-100% depending on the mammal species [19].

These findings prompted us to extend the search for lymnaeid snails across an altitudinal gradient in the Department of Malargüe, with the aim to characterize snail populations using morphological tools and multiple molecular markers, while also describing associated environmental and ecological factors.

## Methods

### Study area

**Figure 1:**
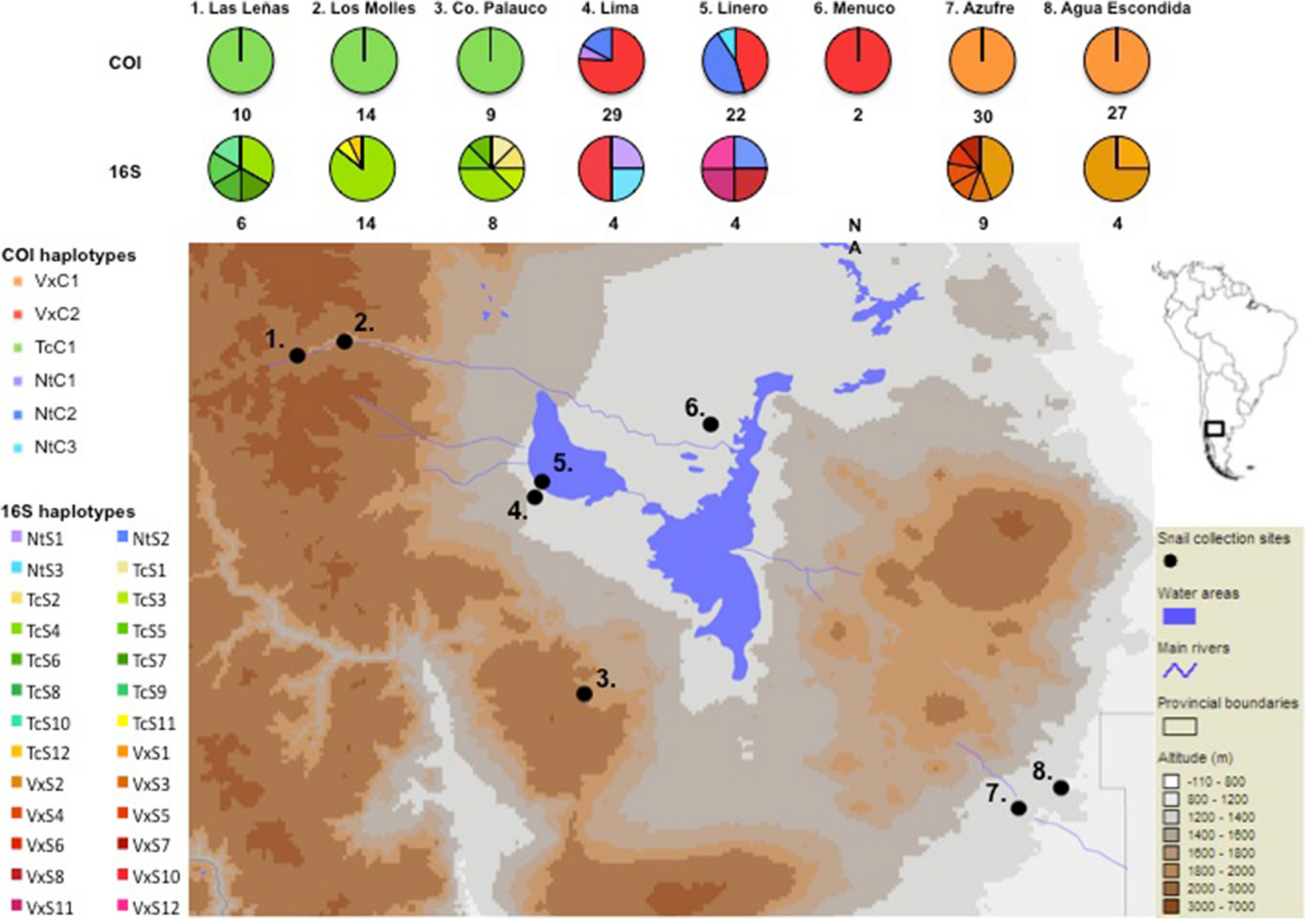
**Map of the study sites, showing landscape altitudinal gradients and diversity of COI and 16S haplotypes at each location.** The haplotypes labeled “Vx” refer to *Galba viatrix*, those labelled “Nt” refer to *G. neotropica* and those labeled “Tc” refer to *G. truncatula*.

The study was performed in the Department of Malargüe, Mendoza Province, Northern Patagonia, Argentina. This region involves areas of pre-cordillera on the west (2000–3500 meters above sea level [masl]) and arid flat plains characterized by dunes and xerophytic vegetation at the east (<1300 masl). This altitudinal gradient is not smooth (Figure 1) and flat plains are surrounded by canyons, volcanoes and eroded hills. The climate is arid and cold to temperate-cold [23]. In the lower flat lands (< 1300 masl) summer is the rainy season and mean annual precipitation is about 300 mm, while in the higher areas (> 1900 masl) precipitation is concentrated mainly in the winter as snowfall [23].

### Snail collection procedure

Snail collection sites were chosen based on the following characteristics: a) markedly different altitudes, b) human activity and c) presence of farm animals and water bodies.

Accordingly, the following eight sites were chosen for snail collection: Las Leñas valleys (2019 masl - S35°11′48.1″; W70°02′20.7″), Los Molles valleys (1950 masl - S35°09′55.4″; W69°56′10.7″), Cerro Palauco in La Payunia natural reserve (1940 masl - S35°57′18.1″; W69°24′55.1″), Lima (1386 masl - S35°30′49″; W69°31′25.7″) and Linero (1386 masl - S35°28′43″; W69°30′26″) near Malargüe town, Los Menucos stream near Laguna de Llancanelo Natural Reserve (1280 masl - S35°35′00.3″; W69°14′20.2″), Azufre village (1240 masl - S36°12′39.6″; W68°28′22.7″) and Agua Escondida village (1202 masl - S36°09′54″; W68°22′52.5″). Linear distance between the extremes sampling points was 241 km.

No accessible water bodies were found between 1400–1800 masl. This is explained by the fact that the pre-cordillera rises up to 3000 masl and descends along a steep slope that ends on a foothill (at approximately 1500 masl) that extends 10–20 km eastwards [24]. This steep slope is associated with significant erosive action, making this area unsuitable for water deposition and thus snail colonization.

Collections of snails were performed seasonally between January 2008 and December 2009 as part of a long-term study on the epidemiology of *F. hepatica* infection in the area (data in preparation). Given that in the southern hemisphere summer occurs between December and April, and winter between June and September, collections were made during one month in each season, namely: February (summer), May (autumn), August (winter) and October (spring). Snails were collected during 30 minutes by the same person using metal scoops and hand-held sieves or by hand-picking. They were transported alive to the laboratory in plastic flasks containing water from the collecting site. Intense snowing prevented snail collections in Las Leñas and Los Molles during winter 2008 and 2009.

In addition, for each survey and at each site pH and height of water column were recorded as well as habitat characteristics such as predominant bottom type (rocky, sandy, muddy), water flow (standing, fast running, and low running), periodicity (temporary, permanent) and origin (natural, man-made). Finally, as *G. viatrix* and *G. truncatula* are truly amphibious snails, which can spend considerable time submerged by only a thin layer of water or even exposed completely on rocks, mean air temperature data were also obtained from the nearest meteorological station. The Kruskal-Wallis non-parametric test [25] was used to compare the mean air temperature among collection sites in each season. For this analysis mean daily air temperatures of the survey date +/- 3 days-interval were used. The Wilcoxon Rank non-parametric test [25] was used to compare the rest of the environmental variables among collection sites.

### Snail identification, parasitological examination and morphological technique

Morphological- and molecular-based identifications were performed on 143 individuals, representing 3% of the total collected snails (n = 4705) during the study period.

For parasitological analysis, snails were placed in plastic vials containing de-chlorinated water and exposed to artificial light to stimulate the shedding of *F. hepatica* cercariae, for at least 2 hours [26].

Afterwards, snails were relaxed overnight in water containing menthol crystals, and then killed in hot water (60° C). Each individual was removed from its shell; a piece of the head-foot was cut off and preserved in 100% ethanol, while the rest of the soft tissue was placed in slightly modified Raillet-Henry fluid [9]. The soft tissue was dissected to reveal the internal anatomy and particularly the male reproductive organ of each snail. Taxonomic identification was based on the ratio between penis sheath length and prepuce length, and the shape and size of the prostate gland [9]. All measurements were made using a stereoscopic microscope with a graduated eyepiece.

### DNA extraction and amplification

Genomic DNA was extracted from the head-foot tissue (preserved in 100% ethanol, as described above) of 143 individual snails using a standard CTAB protocol [27]. Separate PCRs were performed to amplify fragments of the cytochrome oxidase sub-unit 1 (COI) gene and the 16S ribosomal RNA gene (16S), using standard universal primers (LCO1490 and HCO2198 and 16arm/16brm) and cycling conditions [28,29]. Amplifications were done using Promega Go-Taq (Promega Corporation, Madison, UK) in a 25 μl reaction. All successful amplifications were purified using either a Millipore PCR_96_ Cleanup kits on a vacuum manifold (Millipore, Billerica, USA) or ExoSAP-IT (GE Healthcare Biosciences, Pittsburgh, USA) as per manufacturer’s instructions, using pure water for washing and resuspension. Product concentration was quantified on a Nanodrop ND-1000 Spectrophotometer (Nanodrop Technologies Inc., Willington, USA), and sequencing reactions were performed on purified PCR products using an Applied Biosystems Big Dye Kit (version 1.1) and run on an Applied Biosystems 3730 DNA Analyzer (Applied Biosystems, Carlsbad, USA). Sequences were assembled and edited by eye using Sequencher v 4.8 (Gene Codes Corporation, Ann Arbor, Michigan, USA: http://www.genecodes.com) and Geneious v 5.3.6 (Biomatters Ltd., Auckland, New Zealand) and aligned using MUSCLE (http://www.ebi.ac.uk/Tools/msa/muscle/).

### Analysis of genetic sequence data

Overall, 143 snails from the seven collection sites were sequenced for the COI gene, and 49 were sequenced for the 16S gene. The mean corrected genetic distances were calculated for within and between each haplotype as well as each species. The best-fit model of nucleotide substitution for each sequence dataset was determined by comparing model likelihoods based on neighbour-joining trees, using PAUP version 4 [30].

Maximum likelihood-based tree-building was used to investigate the relationships between the mitochondrial haplotypes, using the same model of nucleotide substitution as for the corrected distance estimations. Node support was determined through bootstrapping (1000 replicates in all cases). PhyML [31] was used to create the maximum likelihood tree and subsequent bootstrapping. Trees were visualised and edited using FigTree v 1.3.1 (http://tree.bio.ed.ac.uk/software/figtree/).

## Results

### Morphological diagnosis

Based on morphological taxonomy, *Galba viatrix* and *Galba truncatula* were the two species identified in the study area. For simplicity, throughout the rest of the manuscript, species from this group of lymnaeids will be referred to as *Galba* (as opposed to *Lymnaea* or another generic name), as per the recent recommendation by Correa *et al.*[4]. Out of 143 specimens analyzed, 110 (76.9%) were assigned to *G. viatrix* and 33 (23.1%) to *G. truncatula*.

*Galba viatrix* shows an ovoid and pear-like prostate and a greater penial complex with a sheath/prepuce ratio of 1/1 to 1/3 [23]. *G. truncatula* was characterized by a stomach-shaped prostate that increases in diameter toward the *vas deferens* and a penial sheath/prepuce ratio of 1/4 to 1/5 [23]. Both species are indistinguishable in terms of shell morphology [23]. The shell is brown in colour and its whorls increase in diameter rather slowly. The spire is long, narrow and pointed while the body whorl is poorly developed. The aperture is oval and about half as long as the shell length [32].

None of the snails were found infected with either *F. hepatica* or any other trematode parasite.

### Molecular results and analysis

The molecular results revealed the presence of three, rather than two, species of lymnaied snail: *G. truncatula, G. viatrix* and also *Galba neotropica*. Out of 143 specimens analyzed, 91 (63.6%) were assigned to *G. viatrix*, 33 (23.1%) to *G. truncatul*a and 19 (13.3%) to *G. neotropica.* For the COI marker, 655 characters were included in the haplotype analysis; of these, 520 were constant and 46 of the variable sites were parsimony informative. Only one haplotype (TcC1, Genbank accession number JN872455) was observed for *G. truncatula*, and all of these individuals had also been identified as *G. truncatula* in the morphological analysis, from the sites Las Leñas, Los Molles and Cerro Palauco (Figure 1). Individuals morphologically identified as *G. viatrix* from Agua Econdida, Azufre and Menucos all shared the same haplotype (VxC1, Genbank accession number JN872449), which corresponded to *G. viatrix* after a BLAST search (http://blast.ncbi.nlm.nih.gov/). A second *G. viatrix* haplotype (VxC2, Genbank accession number JN872450) was observed at the sites Lima and Linero.

However, 19 individuals, 7 from Lima and 12 from Linero, earlier identified as *G. viatrix* on the basis of morphology, had COI haplotypes that corresponded to *G. neotropica*; in total, three *G. neotropica* haplotypes were observed (NtC1-NtC3; Genbank accession numbers JN872452-JN872454). While the sympatric species varied in frequency and haplotype composition during the two years that snail collections were made from the sites, the combination of VxC2 and NtC2 was the most frequent. In spring 2009, three different haplotypes were simultaneously collected in Lima, the maximum number observed during this sampling period (Table 1). NtC1 was exclusively found in Lima, always co-occurring with *G. viatrix* (VxC2).

**Table 1:** **Sympatric combination of *Galba viatrix *** **and *Galba neotropica *** **COI haplotypes in Lima and Linero, Mendoza Province, Northern Patagonia, Argentina**

	**Sampling date**						
	**2008**			**2009**			
**Collection site**	**Autumn**	**Winter**	**Spring**	**Summer**	**Autumn**	**Winter**	**Spring**
**Lima**	VxC2	VxC2	VxC2	VxC2	N/A	VxC2	VxC2
	NtC2	NtC1					NtC2
							NtC1
**Linero**	VxC2	VxC2	VxC2			N/A	N/A
		NtC2	NtC2	NtC3	NtC2		

N/A = not analyzed, VxC2 = *G. viatrix*, NtC1 = *G. neotropica*, NtC2 = *G. neotropica*, NtC3 = *G. neotropica.*

For the 16S data, 430 characters were included in the haplotype analysis. Of these, 364 were constant, and 34 of the variable sites were parsimony informative. As with the COI data, the 16S sequences showed division into three species, with *G. truncatula* sequences corresponding to the individuals morphologically diagnosed as *G. truncatula*, and the *G. viatrix* and *G. neotropica* sequences were divided among the individuals morphologically diagnosed as *G. viatrix*. However, the 16S sequences were generally more variable than the COI data, with 12 unique haplotypes observed for the *G. truncatula* individuals (GenBank accession numbers JN872477-JN872488), 11 haplotypes for *G. viatrix* (JN872459-JN872466, JN872468-JN872470) and 3 haplotypes for *G. neotropica* (JN872471-JN872473). Similarly, as with the COI data, the mixed *G. viatrix* and *G. neotropica* sites were Lima and Linero. In comparison to the COI sequences, there was much more intrasite variation with the 16S data, particularly with Cerro Palauco and Azufre, which each had 6 unique 16S haplotypes (of *G. truncatula* and *G. viatrix*, respectively), compared to a single COI haplotype in each. The geographical distribution of the 16S haplotypes can be seen in Figure 1.

**Figure 2:**
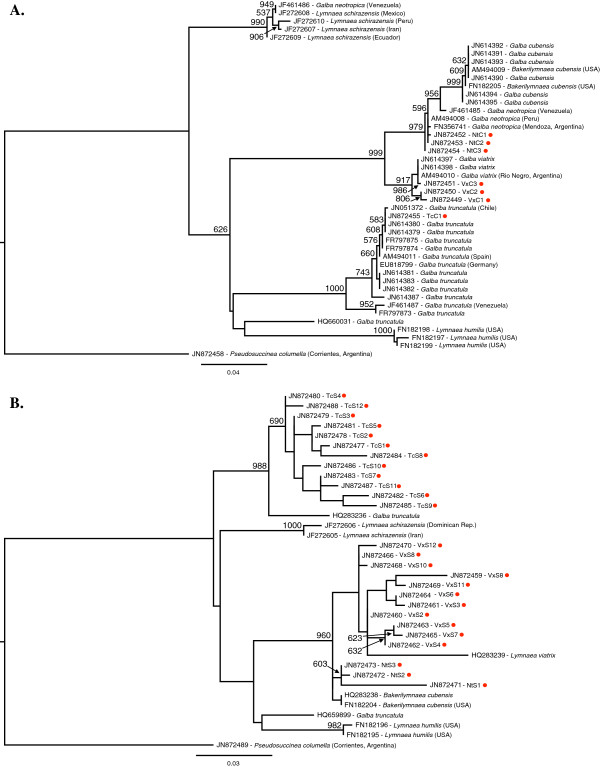
**Phylogenetic trees of the mitochondrial sequence data, including sequences taken from GenBank.** The trees were created using maximum likelihood, and node support values (for those > 500) are based on 1000 bootstrap replicates. Each sequence is shown with its GenBank accession number, followed by the haplotype code (for our sequences) or the species name (as per the GenBank accession information, as of August 28, 2012). If a geographical location was given as part of the GenBank accession information, it is also included, in brackets. Each of the Mendoza sequences we produced for this paper is identified with a red dot. *Pseudosuccinea columella* was used as the outgroup. **A:** COI data. **B:** 16S data.

Maximum likelihood-based phylogenetic analysis of the recovered haplotypes, together with published GenBank sequences of related species, showed that *G. viatrix* and *G. neotropica* had strong support as sister species based on the COI region, but that *Galba* (=*Bakerilymnaea*) *cubensis* also clustered within the *G. neotropica* clade (though monophyletic within it). However, with 16S, while there was strong overall support for the *G. viatrix/neotropica/cubensis* clade, only *G. neotropica* had more than 50% bootstrap support as its own grouping. *G. truncatula* was more distantly related based on both markers, and was monophyletic, apart from one GenBank sequence in each tree which appeared to be misidentified *Lymnaea humilis* (Figure 2A and B).

Corrected distance estimates between and within haplotypes supported the closer genetic relationship between *G. viatrix* and *G. neotropica*, though the COI data showed greater distance from these species to *G. truncatula* than the 16S data (Tables 2 and 3).

**Table 2:** Pairwise distance values between the COI haplotypes from Mendoza

	**VxC1**	**VxC2**	**NtC1**	**NtC2**	**NtC3**	**TcC1**
**VxC1**	-	0.03	0.047	0.046	0.044	0.099
**VxC2**	0.003	-	0.047	0.046	0.044	0.099
**NtC1**	0.054	0.054	-	0.002	0.003	0.105
**NtC2**	0.052	0.052	0.002	-	0.002	0.104
**NtC3**	0.050	0.050	0.003	0.002	-	0.105
**TcC1**	0.140	0.140	0.154	0.152	0.154	-

Values below the diagonal correspond to corrected distances using the GTR + G model of nucleotide substitution, while values above the diagonal are uncorrected p-distances. “Vx” refers to *G. viatrix* haplotypes, “Nt” to *G. neotropica* and “Tc” to *G. truncatula*.

**Table 3:** Average pairwise distance values between the 16S haplotypes from Mendoza

	***G. viatrix***	***G. neotropica***	***G. truncatula***
***G. viatrix***	**0.010**	0.021	0.056
	**(0.000-0.017)**	(0.009-0.031)	(0.045-0.073)
***G. neotropica***	0.021	**0.014**	0.056
	(0.009-0.031)	**(0.002-0.021)**	(0.045-0.071)
***G. truncatula***	0.057	0.058	**0.012**
	(0.046-0.076)	(0.046-0.074)	**(0.000-0.026)**

Values (in bold) along the diagonal represent average within-species distance: there were no differences between corrected and uncorrected values for these means and ranges. Values below the diagonal correspond to corrected distances (using the GTR model of nucleotide substitution), and values above the diagonal are uncorrected p-distances. The values in parentheses indicate the range of values observed.

### Description of lymnaeid habitats

**Figure 3:**
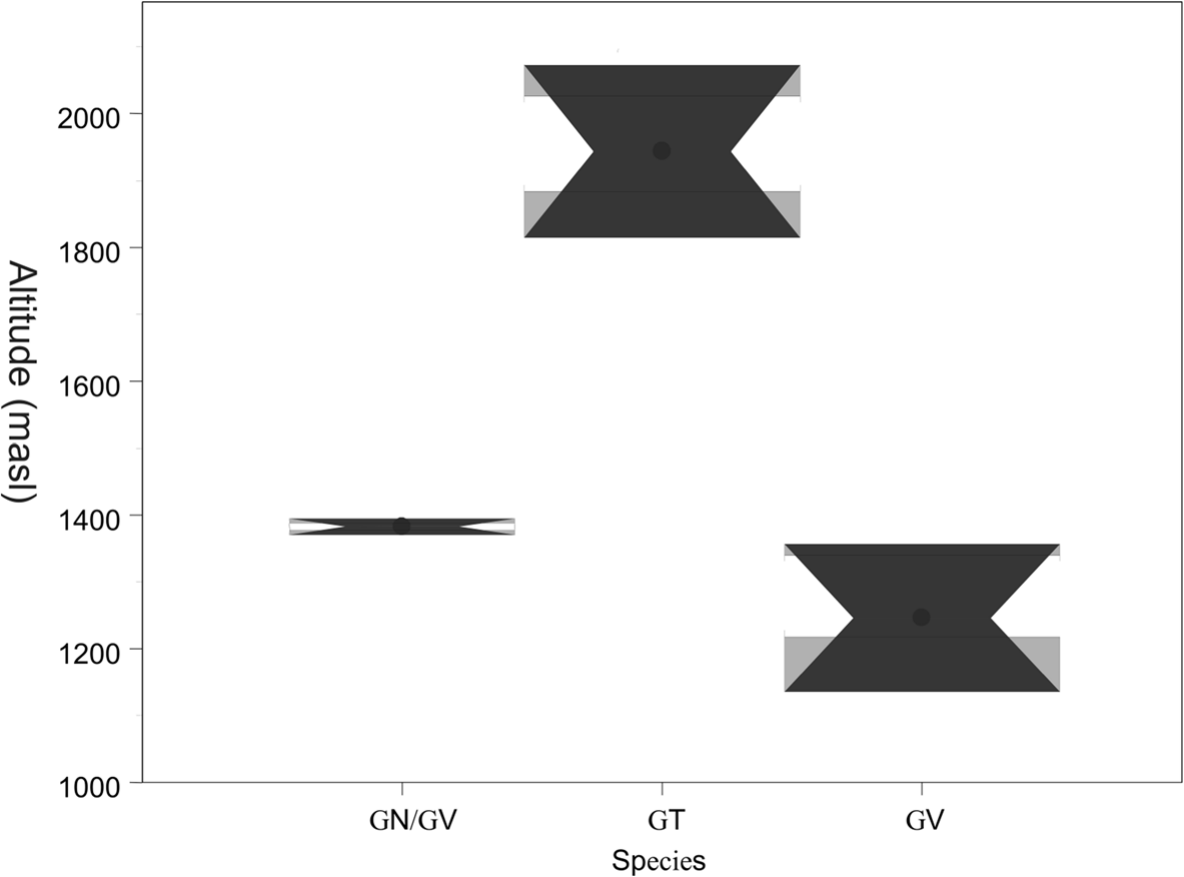
**Box & Whisker plot showing the altitude of the water bodies inhabited by *Galba truncatula *(GT), *Galba viatrix *(GV) and *Galba viatrix/Galba neotropica *(GV/GN).** The square indicates the median value; the box encompasses the 1^st^ and 3^rd^ quartiles and the whisker encompasses the minimum and maximum values.

*G. truncatula, G. viatrix* and *G. neotropica* differed significantly in the altitudes of their habitats (Z = 2.087, p = 0.0369) (Figure 3). *G. truncatula* was found only in the collection sites located above 1900 masl (Las Leñas, Los Molles and Cerro Palauco) while *G. viatrix* and *G. neotropica* were limited to sites located below 1400 masl (Lima, Linero, Agua Escondida, Azufre and Menucos).

The collection sites where *G. viatrix* were found, either alone (VxC1; Agua Escondida, Azufre and Menucos) or in sympatry with *G. neotropica* (VxC2/NtC1-C3; Lima and Linero), displayed similar environmental characteristics.

*G. truncatula* was found in temporal streams and marshes of natural origin, with rocky bottom and shallow and fast running water. Only in Los Molles valley this species was found in swamps of thermal spring standing water. The habitats where *G. viatrix* was found in sympatry with *G. neotropica* were temporary marshes, swamps and ponds of shallow standing water and muddy bottom, all of natural origin.

In turn, the habitats where *G. viatrix* was found alone were the most variable: in Los Menucos permanent shallow streams of fast running water with rocky bottom were observed; in Azufre snails were found in temporary shallow swamps and streams of slow running water and muddy bottom, while Agua Escondida was the only site consisting of an artificial habitat composed of temporary reservoir ponds of standing water for domestic use.

pH values were always alkaline, ranging between 7.3 and 9.4 and showed little variation among sites, seasons and years. Regarding habitat depth, most of the collection sites were shallow water bodies (0–5 cm) with the exception of Lima, Linero and Cerro Palauco where height of water column occasionally reached up to 22 cm.

*Galba viatrix* and *Galba neotropica* collection sites (Lima/Linero and Azufre/Agua Escondida) showed the same temperature pattern, with mean air temperatures between 0 and 10°C in autumn and winter, and between 10 and 20°C in summer and spring. *Galba truncatula* collection sites were highly variable among themselves. Co Palauco showed the greatest variation with the lowest mean air temperature in winter (-1.6'°c), followed by autumn and spring (2.9-13.4°C) and the highest in summer (17.7°C). Las Leñas was the coldest site and Los Molles the warmest site, with mean air temperatures below and above 10°C all year round, respectively. Moreover, Los Molles was the only site with mean air temperature higher than 20°C.

## Discussion

Our study demonstrated the benefit of incorporating environmental variables alongside integrated molecular and morphological diagnosis in the identification of lymnaeid species in Argentina. The morphological analysis suggested the presence of two species (*G. truncatula* and *G. viatrix*) in the sites surveyed, clearly separated by altitude, which would have been consistent with other studies of *Galba*/*Lymnaea* from elsewhere in South America [10]. However, the molecular analysis recovered three distinct species (*G. truncatula*, *G. viatrix* and *G. neotropica*), with a much more complex pattern of distribution across the landscape and altitudinal gradient of the area.

Historically, *G. truncatula* was considered to be an introduced species from Europe [33] where it is the main intermediate host of *Fasciola hepatica*. Nevertheless, a recent phylogenetic analysis suggested that ancestral *G. truncatula* actually originated in the Americas and when it reached Europe, it evolved and diverged from its source population [13]. Its presence in South America has been hypothesized as the result of introductions from this European group; a small founding population, creating a bottleneck, could be a reason for the low genetic variability of *G. truncatula* observed in this work. However, other possibilities, such as high levels of self-fertilization or more recent, environmentally-determined, population bottlenecks should also be considered [34]. For example, this species has been observed typically to inhabit areas located at high altitude such as the Bolivian Altiplano [35] and the Kitulo Plateau in the Southern Highlands of Tanzania [36], though rarely from latitudes as far south as the study sites examined here. Environmental fluctuations at locations over 1900 masl are usually extreme, as observed in Cerro Palauco where mean daily temperature were below 10°C for 6 months (April-October 2009). These temperatures could limit snail biological processes [37] with the possible outcome of demographic instability, though these hypotheses should be tested further.

*Galba viatrix* is common throughout South America [38] and shows the broadest distribution in Argentina, with its southernmost limit in Santa Cruz Province, Andean Patagonia (49°59′ S, 68°55′ W) [39]. *G. viatrix* is the main intermediate host of *F. hepatic*a in Argentina [21,22] and has a remarkable plasticity to adapt to many different habitats and environmental conditions, including artificially made water reservoirs [40]. These national patterns of distribution were mirrored in the present study, with *G. viatrix* showing the widest distribution pattern, inhabiting 4 out of the 7 study sites. It was also the most abundant species, comprising 79.9% of the 143 snails collected, and was found in a variety of different microhabitats, such as marshes, irrigation canals, swamps, ponds, streams and even an artificial reservoir of standing water, highlighting its importance in the region.

Of the three species, *G. neotropica* has received the least research attention, possibly because it is a relatively newly described species. It was first observed in Lima, Peru and thought to be a variety of *Galba (= Lymnaea*) *viatrix*, and named var. B *elongata*[15] and in 2009, molecular studies confirmed its presence in the Department of Luján de Cuyo, Mendoza Province, Argentina [17]. In Mera y Sierra’s work, *G. neotropica* was observed alone at the artificial pond surveyed, and its genetic diversity was limited to only one haplotype. We believe our study is the first to observe *G. neotropica* in sympatry with another lymnaeid species in Argentina, and, moreover, to find multiple haplotypes at more than one locality for the two co-occurring species (*G. neotropica* and *G. viatrix*); recent work (published after our study was conducted) has shown another example of *G. neotropica* mistaken for *G. viatrix* on the basis of morphology, though in that case, *G. viatrix* turned out not to be sympatrically present [41].

So far, and including the present work, *G. neotropica* has been identified only by means of molecular tools. From a morphological perspective, it is indistinguishable from *G. viatrix*. This result raises the question of whether *G. neotropica* is a new and distinct species, or rather a variant or morph of *G. viatrix.* The recognition of the phenotypic variability of *G. viatrix*[35,42] dates to its original description by D’Orbigny [43], when he recognized two varieties (A: *ventricosa* and B: *elongata*). While this species is known to predominately out-cross, which could increase genetic variation and thus increase shell morphological variation [44], many snail species demonstrate impressive shell variation and plasticity independent of their reproductive preferences, and so other underlying genetic, developmental and/or environmental factors may also be implicated [45,46].

The *Galba/Fossaria* group, in which the three species identified in this work are included, is characterized by a marked intraspecific morphological variability and the existence of sibling species [15]. Since a “variety” is not a valid taxonomic entity and cannot be raised to species level, a general consensus is needed to define the taxonomic statuses of species within this group of snails. Any search for consensus implies a generous, open-minded approach that should consider a great range of opinions in order to find constant, apparent differences between biological entities that can separate them into natural groups [47]. Our findings show much greater taxonomic resolution provided by analysis of the COI region as opposed to 16S, suggesting that barcoding of the COI could be a valuable tool for identifying lymnaeids. We also demonstrate the utility of comparing new sequences to existing molecular data, such as that on publically accessible databases such as GenBank, in order to avoid misidentification based on morphology alone. Having said this, it is worth noting the variety of nomenclature observed on databases like GenBank further confounds attempts to reconcile existing species delimitations. Without a conceptual taxonomic framework, ecological studies on lymnaeids, such as examination of population dynamics, spatial distribution, dependence of abiotic conditions and experiments to determine parasite susceptibility, cannot be done.

The observed patterns of genetic variation seen within and between sites and species were a key finding in this study. The genetic variability of *G. viatrix* was clearly evident based on the presence of two different COI haplotypes and 11 16S haplotypes; *G. neotropica* also showed more diversity than observed in other studies [41]. Moreover, different haplotypes were encountered where *G. viatrix* was observed alone as compared to where it occurred in sympatry with *G. neotropica*. The sites where sympatric *G. neotropica* and *G. viatrix* occurred were of middle elevation and located in the ecotonal region between two phytogeographic regions, the Monte and the Patagonica province [48,49]. Ecotonal regions, or ecotones, tend to show higher species diversity due to edge effects [50]; this might also explain the heightened intraspecific genetic variation in Lima and Linero.

More generally, competition or other interactions could have an impact on population sizes, which in turn could affect population structure and haplotypes composition [51]. Further examination of the carrying capacity of these habitats, in conjunction with observations of snail densities in particular niches, could be done to test this hypothesis. Likewise, future studies should test explicitly for evidence of hybridizing between species, which could also account for the observed haplotype composition. Finally, the impact of parasitism should not be overlooked; in many systems, there is evidence for high diversity being a consequence of elevated risk of parasitism, which could be independently driving genetic variation in both species simultaneously [52,53]. Further research is needed to evaluate the possible interactions between *G. viatrix* and *G. neotropica* and their consequences on their parasite-host relationships.

Ecologically, the observation of biologically active snail populations at low temperatures (approx 10°C) deserves particular attention, as this is commonly considered too low to sustain snail metabolism. However, *G. truncatula* is also able to colonize marshes of spring thermal water, which have much higher temperatures, when the occasion arises. At present, limiting factors regulating lymnaeid biology are unknown, though distribution by altitude has been observed in other studies [54]. Temperature and water soil availability were considered the main factors; however, the results obtained in this study open many questions, such as the importance of spatial distribution of species, competition among sibling species and also possible snail population movement along watersheds. Studies aimed at determining spatial distribution, while also factoring in habitat characteristics and temperature ranges suitable for lymneid populations, will be very important in planning future fascioliasis control measures.

As such, integrating this study into a broader examination of fascioliasis transmission in the region will be a crucial next step. All three of the species observed here are known to be susceptible to *Fasciola* infection [17,21-23], so it will be necessary to understand local compatibility with the parasite to see which areas are at highest risk for transmission. As this study was designed to utilize a sub-set of samples from a wider study on *F. hepatica*, we will seek to extend this research and combine the findings with information on fascioliasis in the region.

## Conclusions

This study highlights the need of a robust taxonomic framework for the identification of lymnaeid snails, incorporating molecular, morphological and ecological variables while avoiding nomenclature redundancy. As the three species observed here, including one alien invasive species, are considered hosts of varying susceptibility to Fasciola parasites, and given the economic importance of fascioliasis for livestock production, this research has critical importance for the ultimate aim of controlling disease transmission.

## Competing interests

The authors declare that they have no competing interests.

## Authors’ contributions

CJS performed the molecular work, analysed the data and co-prepared the initial draft of the manuscript and its figures. LP designed and participated in the field study, performed the morphological and environmental analyses and co-prepared the initial draft of the manuscript. SMP and LI assisted with the field work and the morphological and environmental analyses, and commented on the final manuscript. JRS participated in the field work, advised on the molecular analyses and commented on the final manuscript. CW-C designed the study, performed the morphological analyses and co-prepared the initial draft of the manuscript. All authors read and approved the final version of the manuscript.
